# Activation of ERK and p38 Reduces AZD8055-Mediated Inhibition of Protein Synthesis in Hepatocellular Carcinoma HepG2 Cell Line

**DOI:** 10.3390/ijms222111824

**Published:** 2021-10-30

**Authors:** Ha-yeon Jee, Yoon-Gyeong Lee, Sol Lee, Rosalie Elvira, Hye-eun Seo, Ji-Yeon Lee, Jaeseok Han, Kyungho Lee

**Affiliations:** 1 Department of Biological Sciences, Konkuk University, Seoul 05029, Korea; gkdus4806@gmail.com (H.-y.J.); yl97@illinois.edu (Y.-G.L.); sol1667@naver.com (S.L.); tjgpd@naver.com (H.-e.S.); weezel99@naver.com (J.-Y.L.); 2 Soonchunhyang Institute of Medi-Bio Science (SIMS), Soonchunhyang University, Cheonan 31151, Korea; maria.rosalie.elvira@gmail.com (R.E.); hanjs015@sch.ac.kr (J.H.); 3 Korea Hemp Institute, Konkuk University, Seoul 05029, Korea

**Keywords:** 4E-BP1, AZD8055, ERK1/2, mTORC1/2 inhibitor, p38, protein synthesis

## Abstract

Protein synthesis is important for maintaining cellular homeostasis under various stress responses. In this study, we screened an anticancer drug library to select compounds with translational repression functions. AZD8055, an ATP-competitive mechanistic target of rapamycin complex 1/2 (mTORC1/2) inhibitor, was selected as a translational suppressor. AZD8055 inhibited protein synthesis in mouse embryonic fibroblasts and hepatocellular carcinoma HepG2 cells. Extracellular signal-regulated kinase (ERK) and p38 mitogen-activated protein kinase (MAPK) were activated during the early phase of mTORC1/2 inhibition by AZD8055 treatment. Combined treatment of AZD8055 with the MAPK kinase1/2 (MEK1/2) inhibitor refametinib or the p38 inhibitor SB203580 markedly decreased translation in HepG2 cells. Thus, the inhibition of ERK1/2 or p38 may enhance the efficacy of AZD8055-mediated inhibition of protein synthesis. In addition, AZD8055 down-regulated the phosphorylation of eukaryotic initiation factor 4E-binding protein 1 (4E-BP1), and AZD8055-induced phosphorylation of ERK1/2 and p38 had no effect on phosphorylation status of 4E-BP1. Interestingly, AZD8055 modulated the *4E-BP1* mRNA pool by up-regulating ERK1/2 and p38 pathways. Together, these results suggest that AZD8055-induced activation of MAPKs interferes with inhibition of protein synthesis at an early stage of mTORC1/2 inhibition, and that it may contribute to the development of resistance to mTORC1/2 inhibitors.

## 1. Introduction

Regulation of protein synthesis is an energy-requiring cellular process. Several eukaryotic initiation factors (eIFs) tightly regulated by various signals are involved in translation initiation. As dysregulation of translation machinery in cancer cells is associated with poor cancer prognosis, it is imperative to investigate the role of anticancer agents in the regulation of protein synthesis. For instance, eIF4E-binding proteins (4E-BPs) are well-known suppressors of protein synthesis. Hypophosphorylated 4E-BP1 competes with eIF4G and binds to eIF4E, therefore interfering with the formation of the 43S pre-initiation complex and consequently disrupting protein synthesis [1,2]. Phosphorylation at multiple sites of 4E-BP1 is regulated by various protein kinases such as mechanistic target of rapamycin (mTOR) and mitogen-activated protein kinases (MAPKs). Subsequently, hyperphosphorylated 4E-BP1 fails to bind to eIF4E and is released, which restarts the translation machinery [3,4,5,6,7].

MAPKs are serine/threonine protein kinases that activate multiple cellular signaling pathways such as gene expression and programmed cell death through sequential phosphorylation of different molecules. MAPKs include extracellular signal-regulated kinases (ERKs), p38 MAPKs, and c-Jun N-terminal kinases (JNKs) [8]. Given their substrate specificity, each MAPK can bind specifically to its downstream target [9]. ERK1/2 is activated by growth factors and plays an important role in cell proliferation and survival [10]. Dysregulation in the upstream kinase rat sarcoma (Ras) may contribute to ERK-mediated oncogenesis [11]. In contrast, p38s and JNKs, also known as stress-activated protein kinases, mainly activate inflammatory and stress responses [12,13,14,15]. However, the crosstalk between MAPK signaling and activity may vary depending on the cell type and stress conditions [16].

mTOR is a serine/threonine-protein kinase that is distinguished into mTORC1 and mTORC2 by its key components; mTORC1 contains regulatory-associated protein of mTOR (Raptor), while mTORC2 contains rapamycin-insensitive companion of mTOR (Rictor). mTORC1 controls translation initiation and energy metabolism by regulating its downstream targets such as 4E-BP1 and S6K. mTORC2 functions in cell survival and cytoskeleton rearrangement by activating protein kinase B (AKT) or protein kinase C (PKC) [17]. Several studies have reported that the activation of mTOR signaling is common in numerous cancer cells [18]. Its physiological and pathological significance is true because of its multifunctional mechanism.

Therefore, first-generation mTORC1 allosteric inhibitors, rapamycin and its analogs (rapalogs), have been developed [19]. However, the application of these agents to monotherapy is limited because of AKT activation [20,21,22] and incomplete inhibition of 4E-BP1 function [5]. To overcome these problems, second-generation mTOR inhibitors, also known as ATP-competitive mTORC1/2 inhibitors, such as AZD8055, have been developed [23,24]. Many reports have shown that treatment with these drugs completely reduces residual 4E-BP1 Thr37/46 phosphorylation and AKT Ser473 phosphorylation [25,26,27]. These agents exhibit significant antitumor effects in a variety of cancer cells compared to rapamycin [28,29,30].

Recent studies, however, have found that mTORC1/2 inhibitors have side-effects such as restoration of AKT Thr308 phosphorylation [31,32], epidermal growth factor receptor (EGFR) feedback activation [33], or ERK activation, which are more serious than the side-effects related to rapalogs [34,35]. Therefore, it is essential to elucidate the signaling pathway of mTORC1/2 inhibitors to provide an accurate clinical prognosis. Studies have demonstrated ERK1/2 activation mediated by mTORC1/2 inhibitors, but there is little evidence of their effects on other MAPK signaling or protein synthesis. In the present study, we show that in the HepG2 hepatocellular carcinoma cell line, AZD8055-mediated translational inhibition is masked by ERK1/2 and p38 activation. Our studies suggest that AZD8055 exerts an inhibitory effect on the mTORC1/2 downstream pathway and that the AZD8055-mediated activation of ERK1/2 and p38 is likely to increase resistance to mTORC1/2 inhibitors in HepG2 cells.

## 2. Results

### 2.1. The mTOR Inhibitor AZD8055 Was Selected during the Screening Process as an Anticancer Drug with Inhibitory Effects on Protein Translation

**Figure 1:**
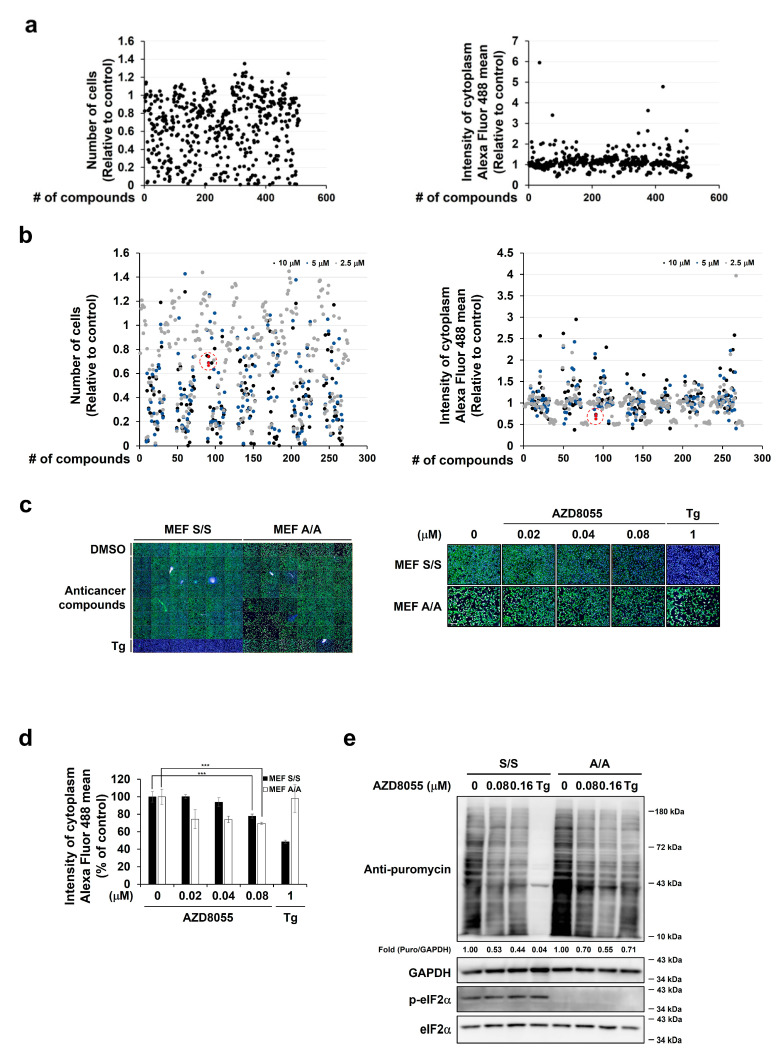
Screening anticancer drugs for identification of translation inhibitors. (**a**,**b**) The level of translation in mouse embryonic fibroblasts (MEFs) was measured using puromycin incorporation assay. Polypeptide chains in wild-type MEFs treated with 10 μM (**a**) or the indicated concentrations (**b**) of Selleck Anti-cancer Compound Library for 24 h were labeled with puromycin for 10 min. The number of cells (left column) and Alexa Fluor 488 intensities of each area (right column) were assessed using Operetta High-Content analysis system. Operetta images were analyzed using Harmony software. Red dots in the red dotted circle in B indicate MEFs treated with various concentrations of AZD8055. To recognize the shape of cells, cells were stained with Hoechst 33342 prior to the measurement of cytoplasmic Alexa Fluor 488 intensities. The number of compounds include DMSO- and Tg-treated samples. (**c**–**e**) Wild-type (S/S) or eIF2α mutant (A/A) MEFs were treated with the indicated concentrations of AZD8055 for 24 h; two representative confocal images are shown (**c**). The intensity of Alexa Fluor 488 was visualized (**c**) and MEFs treated with various concentrations of AZD8055 were magnified (**c**). Scale bar = 200 μM. The obtained images were measured using Harmony Software (**d**). Immunoblot analyses were performed to determine the relative amount of puromycin and phosphorylated form of eIF2α using specific antibodies for puromycin, eIF2α, phosphorylated form of eIF2α (p-eIF2α), and GAPDH (**e**). Treatment with thapsigargin (Tg), an ER stress inducer, was used as a positive control. Fold changes in d were calculated by comparing DMSO control group of each cell line with AZD8055 treatment groups. Statistically significant differences were determined by Student’s *t*-test and were indicated as *** *p* < 0.001.

We attempted to select compounds with inhibitory functions on protein translation. After conducting different trials, the following screening process was performed using an anticancer drug library: MEFs were treated with 10 μM drugs from the Selleck Anticancer Compound Library for 24 h, and the degree of protein synthesis was analyzed using puromycin incorporation assay and Operetta High-Content analysis system. However, as the conditions used were too harsh for the cells, low cell viability affected the level of translation (Figure 1a). A secondary screening test was, therefore, conducted using diluted drugs under the assumption that accurate protein synthesis can be measured under conditions when cell viability is higher than 50%. As a result, AZD8055, an mTOR inhibitor, was found to inhibit protein synthesis without significantly affecting cell viability (Figure 1b). As eIF2α is a well-known translational regulator that responds to various stress stimuli [36,37], we investigated whether AZD8055-mediated translational inhibition is dependent on eIF2α. We used wild-type MEFs with normal eIF2α protein (MEF S/S) and mutant MEFs with mutated eIF2α phosphorylation site (serine 51 was replaced with alanine) (MEF A/A) (Figure 1c). Protein synthesis decreased in thapsigargin (Tg)-treated wild-type MEFs but not in Tg-treated mutant MEFs; however, both wild-type and mutant MEFs treated with AZD8055 showed a significant decrease in protein synthesis (Figure 1c,d). Similar results were observed in the Western blots from the puromycin incorporation assay (Figure 1e). Under these experimental conditions, AZD8055 treatment in wild-type MEFs did not change the phosphorylation of eIF2α (Figure 1e). Tg, used as a positive control for the degree of protein synthesis, had no obvious effect on the phosphorylation of eIF2α because it was used for a short time (1 μM, 30 min). These results suggest that AZD8055 inhibits translation and that the AZD8055-mediated translational inhibition is independent of eIF2α function.

### 2.2. AZD8055 Inhibits Protein Synthesis and Up-Regulates Phosphorylation of ERK1/2 in Hepatocellular Carcinoma HepG2 Cells

**Figure 2:**
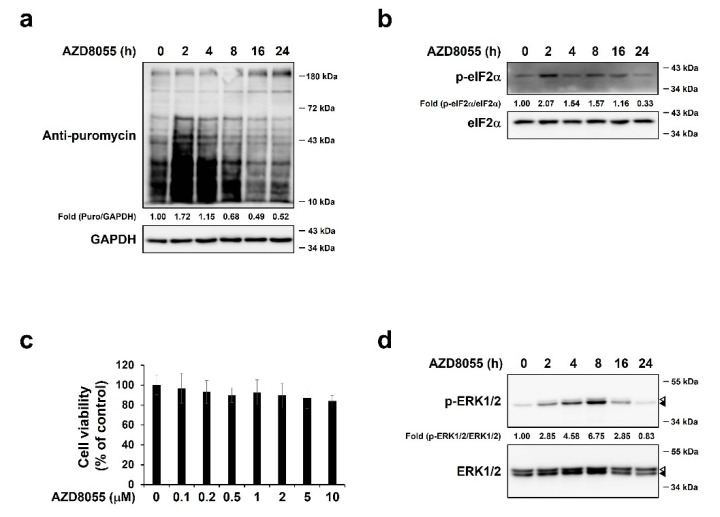
AZD8055 inhibits protein synthesis and up-regulates phosphorylation of ERK1/2 in hepatocellular carcinoma HepG2 cells. (**a**,**b**) HepG2 cells were treated with 2 μM AZD8055 for the indicated periods. Cells were pulse-labeled with puromycin for 10 min and immunoblot analyses were performed. The relative levels of puromycin and p-eIF2α were normalized using GAPDH (**a**) and eIF2α (**b**), respectively. (**c**) MTT assay was performed to measure viability of cells treated with various concentrations of AZD8055 for 24 h. The data obtained from three independent experiments were presented as means ± S.D. (**d**) HepG2 cells were treated with 2 μM AZD8055 for 2–24 h and immunoblot analyses were performed using specific antibodies for ERK1/2 and phosphorylated form of ERK1/2 (p-ERK1/2). Open arrowheads and closed arrowheads indicate ERK1 and ERK2, respectively.

As cancer cells actively produce anti-apoptotic proteins rather than pro-apoptotic proteins [38,39], we attempted to understand the effect of AZD8055 on translation in cancer cells using the hepatocellular carcinoma cell line HepG2. HepG2 cells treated with 2 μM AZD8055 for 2–24 h were pulse-labeled with puromycin. The relative intensity of puromycin increased at 2 h and then started to decrease from 8 h (Figure 2a). The phosphorylation of eIF2α was up-regulated under these conditions (Figure 2b), but the pattern was not consistent with AZD8055-mediated regulation of translational. These results were obtained under conditions wherein AZD8055 treatment did not affect cell viability (Figure 2c). Therefore, we concluded that AZD8055 could inhibit protein synthesis in HepG2 cells and aimed to determine the underlying signaling pathways. Recent studies have reported that the resistance to second-generation mTORC1/2 inhibitors is associated with ERK1/2 activation [22,34,35,40]. Therefore, we hypothesized that ERK1/2 activation is related to protein synthesis pathways regulated by AZD8055. We treated HepG2 cells with AZD8055 for 2–24 h and found that the phosphorylation level of ERK1/2 clearly increased, peaked at 8 h, and then decreased at 24 h (Figure 2d) when protein synthesis decreased (Figure 2a). Thus, ERK1/2 activation may be involved in the regulation of AZD8055-mediated protein synthesis.

### 2.3. AZD8055 Up-Regulates Phosphorylation of ERK1/2 and p38 and Independently Down-Regulates Phosphorylation of 4E-BP1

**Figure 3:**
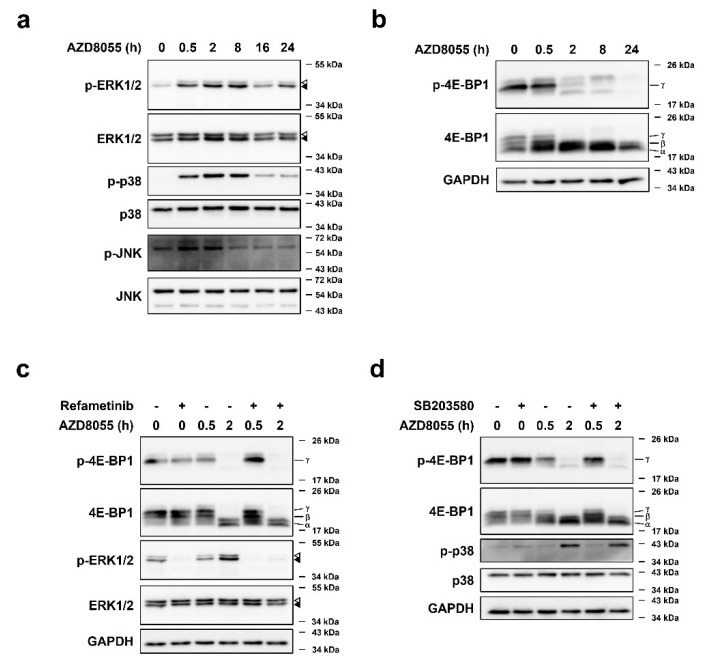
AZD8055 up–regulates phosphorylation of ERK1/2 and p38 and independently downregulates phosphorylation of 4E–BP1. (**a**) HepG2 cells were treated with 2 μM AZD8055 for various time points and immunoblot analyses were performed to measure the activation of three MAPKs using specific antibodies for ERK1/2, p–ERK1/2, p38, p–p38, JNK, and p–JNK. (**b**–**d**) HepG2 cells were treated with 2 μM AZD8055 for various time points (**b**) or pre–treated with or without 100 nM refametinib (**c**) or 10 μM SB203580 (**d**) for 1 h, followed by 2 μM AZD8055 treatment as indicated. Immunoblot analyses were performed using specific antibodies for 4E-BP1, phosphorylated form of 4E–BP1 at Ser65 (p–4E–BP1), ERK1/2, p–ERK1/2, p38, p–p38, and GAPDH. Three isoforms of 4E–BP1 are indicated (α, β, and γ). α and β isoforms are hypophosphorylated, and γ isoform is hyperphosphorylated. Open arrowheads and closed arrowheads indicate ERK1 and ERK2, respectively.

Next, we investigated whether other MAPKs, p38 and JNK [8], exert similar effects. HepG2 cells were treated with 2 μM AZD8055 and MAPK phosphorylation was detected by immunoblot analyses. Although both p38 and JNK were phosphorylated by AZD8055 treatment, the phosphorylation pattern of p38, but not JNK, was regulated in a manner similar to that of ERK1/2 (Figure 3a).

Several previous studies have shown that ERK1/2 and p38 phosphorylate 4E-BP1, a key factor in protein translation that functions downstream of mTOR [5]. We investigated whether activation of ERK1/2 and p38 by AZD8055 induces phosphorylation of 4E-BP1. Treatment of HepG2 cells with AZD8055 for 0.5–24 h led to a clear decrease in the phosphorylated form of 4E-BP1 at Ser65 (p-4E-BP1) at 2 h; phosphorylated 4E-BP1 almost disappeared thereafter (Figure 3b). This result is almost in line with the decrease in protein synthesis after 8 h of AZD8055 treatment (Figure 2a). Interestingly, the phosphorylation patterns of ERK1/2 and p38 (Figure 3a) did not correlate with those of 4E-BP1. Furthermore, the phosphorylation status of 4E-BP1 in AZD8055-treated HepG2 cells did not change after pre-treatment with the ERK1/2 inhibitor refametinib or the p38 inhibitor SB203580 for 1 h before AZD8055 treatment (Figure 3c,d). These observations suggest that AZD8055 up-regulates phosphorylation of ERK1/2 and p38 but down-regulates that of 4E-BP1, and that the phosphorylation status of 4E-BP1 is unaffected by AZD8055-mediated activation of ERK1/2 and p38.

### 2.4. AZD8055-Mediated ERK1/2 Up-Regulation Was Associated with AZD8055-Mediated Inhibition of Protein Synthesis

**Figure 4:**
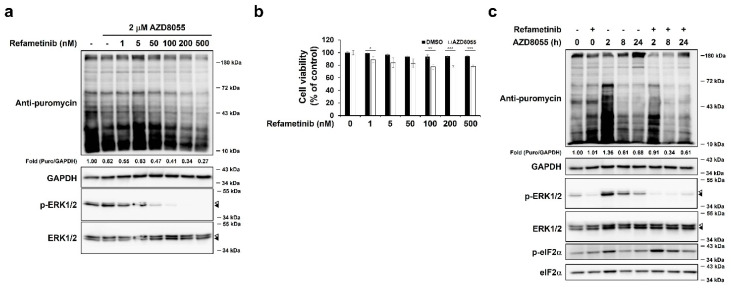
Inhibition of ERK1/2 enhances the efficacy of AZD8055–mediated inhibition of protein synthesis. (**a**) HepG2 cells pre–treated with various concentrations of the MEK1/2 inhibitor refametinib for 1 h were treated with 2 μM AZD8055 for 8 h by adding an AZD8055 solution, and the relative rate of protein synthesis was determined by immunoblotting using specific antibodies for puromycin, ERK1/2, p–ERK1/2, and GAPDH. (**b**) HepG2 cells were cotreated with various concentrations of refametinib and 2 μM AZD8055 for 8 h, and cell viability were measured using MTT assay. The data obtained from three independent experiments were presented as means ± S.D. Statistically significant differences were calculated using Student’s *t*-test and indicated as * *p* < 0.05, ** *p* < 0.01, and *** *p* < 0.001. (**c**) HepG2 cells pre–treated with or without 100 nM refametinib for 1 h were treated with 2 μM AZD8055 for indicated periods. Cell were labeled with puromycin for 10 min and immunoblot analysis was performed using specific antibodies for puromycin, GAPDH, ERK1/2, p–ERK1/2, eIF2α, and p–eIF2α. The relative levels of puromycin were normalized using GAPDH. Fold changes were used to compare the relative translation levels between AZD8055 treated–HepG2 cells with or without refametinib treatment. Open arrowheads and closed arrowheads indicate ERK1 and ERK2, respectively.

Next, we investigated the role of MAPKs in AZD8055-mediated inhibition of protein synthesis. First, we used the MEK1/2 inhibitor refametinib. As MEK1/2 is known to be directly upstream of ERK1/2 [9,41], refametinib treatment significantly reduced ERK1/2 phosphorylation (Figure 4a). Furthermore, refametinib pre-treatment before AZD8055 exposure led to a dose-dependent synergistic effect on translational inhibition (Figure 4a). Indeed, the combined treatment with AZD8055 and refametinib for different time points resulted in lower translational activity than AZD8055 treatment alone (Figure 4c). Under these conditions, however, the phosphorylation of ERK1/2 significantly reduced and that of eIF2α was not relevant to the relative level of puromycin (Figure 4c). Indeed, MEK1/2 inhibitors are known to phosphorylate eIF2α and therefore inhibit translation [42]. Although HepG2 cells are known to be sensitive to MEK inhibitors [31], we used MEK inhibitor concentrations that did not significantly affect cell viability while ERK activity was rapidly inhibited (Figure 4b). These results suggest that early activation of ERK1/2 by AZD8055 positively regulates translation.

### 2.5. Activation of p38 by AZD8055 Treatment Was Associated with AZD8055-Mediated Inhibition of Protein Synthesis

**Figure 5:**
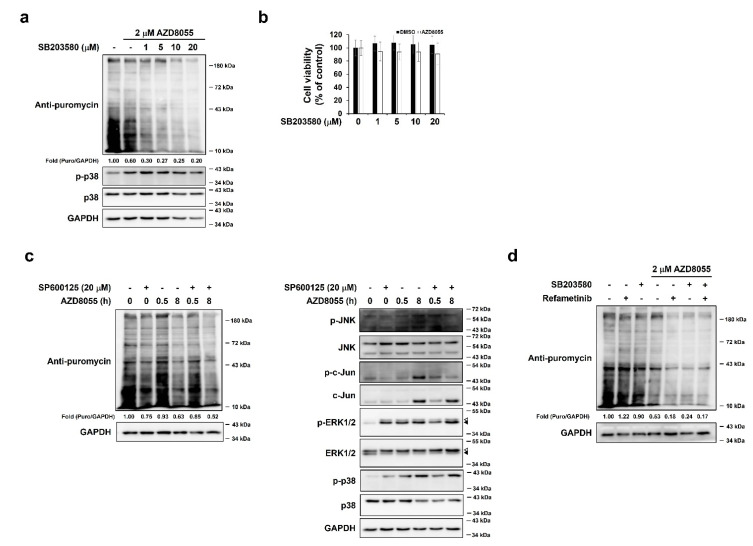
Inhibition of p38, but not JNK, enhances the efficacy of AZD8055–mediated inhibition of protein synthesis. (**a**) HepG2 cells pre–treated with various concentrations of p38 inhibitor SB203580 for 1 h were treated with 2 μM AZD8055 for 8 h and labeled with puromycin for 10 min. Immunoblot analyses were performed using specific antibodies for puromycin, p38, p–p38, and GAPDH. Fold change was determined by comparing DMSO control and SB203580–treated groups. (**b**) HepG2 cells were cotreated with various concentrations of SB203580 and 2 μM AZD8055 for 8 h and MTT assay was performed. (**c**) HepG2 cells pre–treated with or without the JNK inhibitor SP600125 (20 μM) for 2 h were treated with 2 μM AZD8055 for various periods and labeled with puromycin for 10 min. Immunoblot analyses were performed using specific antibodies for puromycin and GAPDH (left), JNK, p–JNK, c–Jun, p–c–Jun, ERK1/2, p–ERK1/2, p38, p–p38, and GAPDH (right). (**d**) HepG2 cells pretreated with or without 100 nM refametinib and 10 μM SB203580 were treated with 2 μM AZD8055 for 8 h. Cells were labeled with puromycin for 10 min and immunoblot analyses were performed using specific antibodies for puromycin and GAPDH. Fold change was determined by comparing the DMSO control group with the inhibitor–treated group. Open arrowheads and closed arrowheads indicate ERK1 and ERK2, respectively.

As the phosphorylation of p38 was regulated in the same manner as that of ERK1/2 by AZD8055 treatment, we investigated whether p38 had a similar effect. Indeed, inhibition of p38 using the inhibitor SB203580 [13] synergistically increased the inhibitory effect of AZD8055 on translation at 8 h in a dose-dependent manner (Figure 5a). As with refametinib treatment, we used SB203580 concentrations and treatment times that did not affect cell viability (8 h; Figure 5b). In contrast, treatment with the JNK inhibitor SP600125 was insufficient to strengthen AZD8055-mediated inhibition of protein synthesis (Figure 5c, left). Concentrations of SP600125 that used in this experiment were sufficient to inhibit the JNK downstream factor c-Jun and had no effect on the phosphorylation of ERK1/2 and p38 (Figure 5c, right). Therefore, the activity of JNK appears to be independent of AZD8055-mediated translational inhibition. Afterwards, we investigated whether ERK1/2 and p38 activation independently affected protein synthesis. Combined treatment of 2 μM AZD8055 with 100 nM refametinib and 10 μM SB203580, however, did not significantly down-regulate the degree of protein synthesis compared to the use of 2 μM AZD8055 with refametinib or SB203580 (Figure 5d). Taken together, these results suggest that AZD8055-mediated up-regulation of ERK1/2 and p38 activation eventually interrupts with AZD8055-mediated inhibition of protein synthesis. In addition, activation of ERK1/2 and p38 by AZD8055 may be integrated into unknown signaling pathways.

### 2.6. AZD8055 Regulates the 4E-BP1 mRNA Pool by Up-Regulating ERK1/2 and p38 Pathways

**Figure 6:**
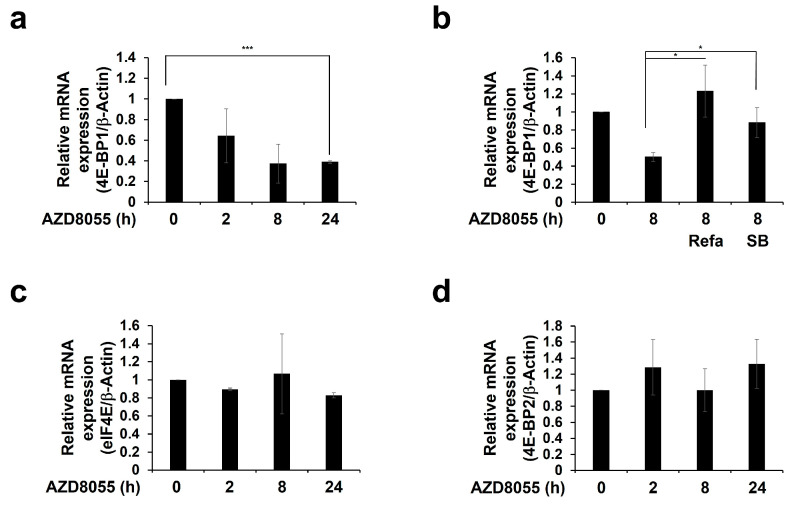
AZD8055 regulates the 4E–BP1 mRNA pool by up-regulating ERK1/2 and p38 pathways. (**a**,**b**) HepG2 cells pretreated with (**b**) or without (**a**) 100 nM refametinib (Refa) or 10 μM SB203580 (SB) for 1 h were treated with 2 μM AZD8055 for indicated periods, and the relative mRNA expression of *4E–BP1* was determined by qPCR using specific primer. (**c**,**d**) Cells were treated with 2 μM AZD8055 for 2–24 h. Endogenous levels of eIF4E (**c**) and *4E–BP2* (**d**) mRNAs were measured by qPCR. The relative expression levels of target mRNAs were determined by comparing DMSO control with the experimental group after normalization using *β–Actin* mRNA. The data obtained from three independent experiments were presented as means ± S.D. Statistically significant differences were calculated using Student’s *t*-test and indicated as * *p* < 0.05 and *** *p* < 0.001.

A previous study has reported a novel paradigm involving ERK1/2- and p38-mediated inhibition of the transcriptional and translational levels of 4E-BP1 [43]. Therefore, we tested whether ERK1/2 and p38 activated by AZD8055 regulate 4E-BP1 transcriptional levels. Treatment with 2 μM AZD8055 reduced the expression of 4E-BP1 mRNA in a time-dependent manner (Figure 6a). Pre-treatment with refametinib or SB203580 prior to AZD8055 rescued the decrease in 4E-BP1 mRNA expression, suggesting that AZD8055-mediated down-regulation of 4E-BP1 mRNA expression is dependent on the ERK1/2 and p38 signaling pathways (Figure 6b). Subsequently, we investigated other factors that are known to be associated with 4E-BP1 function. First, we measured the transcription of eIF4E which is known to decrease hypophosphorylated 4E-BP1 to maintain homeostasis following down-regulation of eIF4E mRNA expression [44]. This phenomenon is also related to the acquisition of resistance [45]. However, we did not observe any significant change in the relative eIF4E mRNA expression level (Figure 6c). Second, 4E-BP2 mRNA, which is known to be ubiquitously expressed and particularly highly expressed in the brain [46,47], was not altered after AZD8055 treatment (Figure 6d). These results suggest that AZD8055 regulates 4E-BP transcription levels via ERK1/2 and p38, which are involved in the inhibition of protein synthesis pathways.

## 3. Discussion

In this study, we screened an anticancer drug library to select compounds with translation-repressive functions. AZD8055, an mTORC1/2 inhibitor, was selected based on its ability to inhibit protein synthesis in MEFs and HepG2 cells without significantly affecting their viability. We investigated factors involved in AZD8055-mediated inhibition of protein synthesis and found phosphorylation of ERK1/2 and p38 to play a positive role in translation. AZD8055 treatment induced phosphorylation of ERK1/2 and p38, and the inhibition of ERK1/2 or p38 led to an increase in the AZD8055-mediated inhibition of protein synthesis. In addition, AZD8055 down-regulated the phosphorylation of 4E-BP1, and the AZD8055-mediated up-regulation of ERK1/2 and p38 phosphorylation was independent of 4E-BP1 phosphorylation status. Interestingly, AZD8055 regulated the 4E-BP1 mRNA pool by up-regulating the ERK1/2 and p38 pathways. Therefore, the results of this study suggest that activation of ERK1/2 and p38 may contribute to the development of resistance to mTORC1/2 inhibitors with antitumor effects.

Studies on translational regulators of cancer cells to enhance anticancer therapy are ongoing [2,48,49] and AZD8055 is one of the known translation suppressors in acute myeloid leukemia cells [30]. Many studies have reported that the mechanism of resistance to mTORC1/2 inhibitors is related to ERK1/2 activation [22,34,35,40]. We tested the role of ERK1/2 in AZD8055-mediated inhibition of protein synthesis and found no inhibitory effect on protein synthesis following single treatment with refametinib; however, co-treatment with AZD8055 and refametinib could significantly down-regulate the protein synthesis pathway after 8 h (Figure 4a,c), a period when ERK1/2 phosphorylation was clearly observed after AZD8055 treatment alone. After 24 h of treatment with AZD8055, ERK1/2 phosphorylation reduced. During this period, the decrease in protein synthesis by co-treatment with AZD8055 and refametinib was not significantly different from that observed after treatment with AZD8055 alone, suggesting that down-regulation of ERK1/2 activity enhances AZD8055 function (Figure 4c). Although the MEK inhibitor used for monotherapy is known to repress eIF2α-mediated translation [42], no inhibitory effect was observed under our experimental conditions (Figure 4c, lane 2). Thus, inhibition of ERK1/2 in mTORC1/2-resistant cells may enhance the effectiveness of chemotherapy. However, there is a slight change in cell viability by AZD8055 treatment compared to its ability to inhibit ERK1/2 activation. This is because the concentration of refametinib used in this experimental condition was low and the treatment time was too short to observe an obvious cell death effect. Several reports have demonstrated that the use of anticancer drugs in combination with refametinib has a synergistic cell death effect both in vitro and in vivo [50,51,52]. Taken together, our results explain why treatment with mTORC1/2 inhibitors and MEK1/2 inhibitors down-regulates cell proliferation and enhances apoptosis [31,39,53].

The MAPK cascade is one of the most important pathways regulating cellular responses, and most studies have noted that ERK1/2 and p38 function distinctly in response to stress signals. Activation of p38 and ERK1/2 under stress conditions occurs differently [8,15,54,55,56]. However, treatment with AZD8055 for a short period activated both ERK1/2 and p38 in HepG2 cells (Figure 3a). In terms of genetics, the neuroblastoma RAS viral oncogene homolog (NRAS) mutant cell line has a disrupted negative feedback loop between ERK1/2 and p38. Consequently, p38 is activated together with ERK1/2 upon anticancer drug treatment, contributing to cell proliferation [16]. Indeed, unlike other HCC cell lines such as Hep3B and Huh-7, HepG2 cells used in the present study carry NRAS mutations [31,50]. Therefore, the results of phosphorylation of both ERK and p38 by AZD8055 treatment are consistent with these reports. In other words, there is limits to obtaining the same results in other cell lines due to the characteristics of HepG2 cells. In addition, as in the case of ERK1/2, inhibition of p38 with AZD8055 treatment resulted in the synergistic down-regulation of protein synthesis at 8 h (Figure 5a). Therefore, contrary to the reports that constitutive ERK1/2 activity and repression of p38 contribute to cell survival through expression of the proto-oncogene transcriptional regulators YAP1 and c-MYC [55], the simultaneous activation of ERK1/2 and p38 observed in our study appears to contribute to cell survival.

Several studies have demonstrated that phosphorylation of 4E-BP1 at multiple sites is mediated by mTOR and MAPKs [4,5,57]. Phosphorylation states and their relative ratios to eIF4E are closely related to the expression of eIF4E-sensitive mRNAs [2,58] and serve as an indicator of the efficiency of ATP-competitive mTORC1/2 inhibitors [59,60,61]. Indeed, treatment with AZD8055 substantially reduced the γ-isoform of 4E-BP1 by inhibiting the phosphorylation of 4E-BP1 at Ser65 (Figure 3b). Therefore, the α-and β-isoforms of 4E-BP1 could bind to eIF4E and inhibit protein synthesis. In addition, the combined treatment with AZD8055 and refametinib or SB203580 did not affect the phosphorylation pattern of 4E-BP1 (Figure 3c,d). Considering that the ERK1/2-mediated 4E-BP1 phosphorylation is induced by growth factors or ERK activators such as phorbol ester [7,62], our results suggest that the AZD8055-induced MAPK activation and reduction of 4E-BP1 phosphorylation in HepG2 cells are mediated via independent pathways.

An increase in translation can be expected from the decrease in the level of 4E-BP1, which acts as an inhibitor of eIF4E. 4E-BP1 is a major target of mTORC1 and is regulated by the degree of phosphorylation [17]. Rolli-Derkinderen et al. showed another regulatory pathway where activated ERK1/2 and p38 down-regulated *4E-BP1* mRNA expression [43]. Consistent with these results, we found that AZD8055 treatment significantly reduced the expression of *4E-BP1* mRNA (Figure 6a). However, the apparent decrease in *4E-BP1* mRNA level at 8–24 h is not consistent with the results shown in Figure 2a describing the decrease in protein synthesis at the same time point. Considering that the 4E-BP1 protein has a long half-life [63,64], we speculate that reduced *4E-BP1* mRNA levels by 24 h may not affect the translation machinery. However, prolonged treatment with AZD8055 may eventually down-regulate 4E-BP1 protein levels. As the eIF4E/4E-BP ratio is an important determinant of the sensitivity to mTOR inhibitors [61], restoration of *4E-BP1* mRNA expression by refametinib or SB203580 treatment is indicative of the restoration of sensitivity to the mTORC1/2 inhibitor (Figure 6b). Thus, our results suggest that ERK and p38 are closely related to the translation machinery through the regulation of *4E-BP1* mRNA expression. Taken together, combination therapy of mTORC1/2 inhibitors and MEK1/2 inhibitors has the potential to synergistically promote cell death.

Although eIF4E overexpression is common in malignant tumors [65], changes in its transcriptional or protein level have little effect on translation [44,49]. Cope et al. showed that *eIF4E* gene amplification confers acquired resistance to AZD8055 and MEK1/2 inhibitors [45]; therefore, we investigated the association between AZD8055-mediated regulation of protein synthesis and eIF4E levels but failed to detect any in HepG2 cells (Figure 6c). Unlike other studies [43], *4E-BP2* mRNA expression did not change (Figure 6d). Based on the roles of ERK1/2 and p38 in AZD8055-mediated protein synthesis, as shown in this study, it seems that enhancing the inhibitory effect on protein synthesis using MEK1/2 inhibitors or p38 MAPK inhibitors would help overcome resistance to mTORC1/2 inhibitors.

## 4. Materials and Methods

### 4.1. Reagents

Thapsigargin (Tg), puromycin dihydrochloride, p38 inhibitor (SB203580), and JNK inhibitor (SP600125) were purchased from Sigma (St. Louis, MO, USA). AZD8055 and refametinib were procured from Cayman Chemical (Ann Arbor, MI, USA). The anti-puromycin and anti-glyceraldehyde 3-phosphate dehydrogenase (GAPDH) antibodies were purchased from Millipore (Burlington, MA, USA) and Santa Cruz Biotechnology (Dallas, TX, USA), respectively. The anti-phospho 4E-BP1 (Ser65), anti-4E-BP1, anti-phospho ERK 1/2, anti-ERK 1/2, anti-phospho p38, anti-p38, anti-phospho-JNK, anti-JNK, anti-phospho eIF2α, anti-eIF2α, anti-phospho c-Jun, anti-c-Jun, and horseradish peroxidase-conjugated secondary antibodies were obtained from Cell Signaling Technology (Danvers, MA, USA). Alexa Fluor^®^ 488 AffiniPure Goat Anti-Mouse IgG (Fcγ fragment specific) was supplied by Jackson ImmunoResearch Laboratories (West Grove, PA, USA). The Selleck Anti-Cancer Compound Library consisting of 414 drugs was purchased from the Department of Convergence Medicine, ASAN Medical Center, University of Ulsan College of Medicine (Seoul, Korea).

### 4.2. Cell Lines and Cell Culture

HepG2 cells were purchased from the American Type Culture Collection (ATCC) and cultured in 10% serum-containing DMEM as previously described [66]. Mouse embryonic fibroblasts (MEFs) eIF2α S/S and MEFs eIF2α A/A were prepared and cultured as described previously [67]. MEFs (eIF2α S/S and eIF2α A/A) were cultured in 10% serum-containing DMEM medium containing MEM amino acid solution (50×, Gibco) and MEM non-essential amino acid solution (100×, Gibco).

### 4.3. Puromycin Incorporation Assay and Analysis

3 × 10^3^ MEFs were plated in 96-well plates a day before treatment with chemicals. The cells were treated with dimethyl sulfoxide (DMSO; Biosesang, Korea) or Selleck Anti-Cancer Compound Library for 24 h. Cells treated with 1 µM Tg for 30 min served as positive control. The medium was replaced with that containing 12 μg/mL puromycin, and the cells were incubated for 10 min. The cells were then fixed with 4% paraformaldehyde for 15 min. After washing twice with phosphate-buffered saline (PBS), the cells were permeabilized in 0.3% Triton X-100 in 1% bovine serum albumin (BSA)/PBS for 45 min and treated with the primary anti-puromycin antibody (1:5000) at 4 °C overnight. After washing with PBS, the cells were incubated with Alexa Fluor^®^ 488 AffiniPure Goat Anti-Mouse IgG (1:400; Alexa Fluor 488-conjugated anti-mouse antibody) for 90 min. Cells were washed with PBS and incubated with Hoechst 33342-containing PBS (1:2500; Invitrogen; Thermo Fisher Scientific, Inc., Waltham, MA, USA). The fluorescence intensity of Alexa 488 was detected using a fluorescence confocal microscope (Operetta High-content analysis system; PerkinElmer, Waltham, MA, USA). The cell number and cytoplasmic intensity of Alexa Fluor 488 in the acquired images were determined using Harmony software.

### 4.4. Measurement of Cell Viability

7 × 10^3^ MEFs or 3 × 10^4^ HepG2 cells seeded in 48-well plates were incubated overnight and then treated with various concentrations of AZD8055. Cells were co-treated with refametinib at different concentrations, as needed. The medium was replaced with fresh medium containing 0.5 mg/mL 3-[4, 5-dimethylthiazol-2-yl]-2, 5 diphenyl tetrazolium bromide (MTT) (Biosesang, Seongnam, Korea) reagent, and the plates were incubated for 30 min (HepG2 cells) or 2 h (MEFs) at 37 °C. The precipitated formazan crystals were dissolved in DMSO, and absorbance was measured using a UVM340 plate reader (ASYS Hitech, Eugendorf, Austria) at a wavelength of 570/690 nm.

### 4.5. Immunoblot Analysis

25 × 10^4^ MEFs or 7 × 10^5^ HepG2 cells were seeded in 60 pi dish the day before chemical treatment. Immunoblot analysis was performed as previously described [68]. The primary antibody (1:1000) and the horseradish peroxidase-conjugated secondary antibody (1:2500) were diluted in 1% skim milk.

### 4.6. Reverse-Transcription Polymerase Chain Reaction (RT-PCR) and Real-Time Quantitative PCR (qPCR) Primers

RT-PCR and qPCR were performed as previously described [69]. The following primer sequences were used to amplify specific genes: Human 4E-BP1, 5′-TCGTGAACACCAGCAGATACC-3′ (forward) and 5′-GTTCTTGTCCACTTCCTGGC-3′ (reverse); human eIF4E, 5′-CCTACAGAACAGATGGGCACTC-3′ (forward) and 5′-GCCCAAAAGTCTTCAACAGTATCA-3′ (reverse) [70]; human 4E-BP2, 5′-TCAAGGCAACTGGTGAAGGG-3′ (forward) and 5′-TCGCTCAAGGGGAATGCAAA-3′ (reverse); human β-Actin, 5′-CATGTACGTTGCTATCCAGGC-3′ (forward) and 5′-CTCCTTAATGTCACGCACGAT-3′ (reverse). The expression level of β-Actin was used as an endogenous control for normalization.

### 4.7. Statistical Analysis

The values in the figures are expressed as mean ± standard deviation (S.D.). The results are representative of at least three independent experiments. Statistical analysis of the data between experimental groups was performed using the Student’s *t*-test. Statistical significance was set at *p* < 0.05.

## 5. Conclusions

- Anticancer drugs that inhibit protein synthesis were selected in MEFs through screening using puromycin incorporation assay.
- AZD8055 inhibits translation in hepatocellular carcinoma HepG2 cell lines.
- AZD8055 inhibits 4E-BP1 phosphorylation and induces ERK1/2 and p38 phosphorylation, which are independent of each other.
- Combined treatment of AZD8055 with refametinib or SB203580 has a synergistic effect on translational inhibition than AZD8055 treatment alone.

## References

[1] Martin D., Nguyen Q., Molinolo A., Gutkind J.S. (2014). Accumulation of dephosphorylated 4EBP after mTOR inhibition with rapamycin is sufficient to disrupt paracrine transformation by the KSHV vGPCR oncogene. Oncogene.

[2] Bhat M., Robichaud N., Hulea L., Sonenberg N., Pelletier J., Topisirovic I. (2015). Targeting the translation machinery in cancer. Nat. Rev. Drug Discov..

[3] Gingras A.C., Raught B., Gygi S.P., Niedzwiecka A., Miron M., Burley S.K., Polakiewicz R.D., Wyslouch-Cieszynska A., Aebersold R., Sonenberg N. (2001). Hierarchical phosphorylation of the translation inhibitor 4E-BP1. Genes Dev..

[4] Liu G., Zhang Y., Bode A.M., Ma W.Y., Dong Z. (2002). Phosphorylation of 4E-BP1 is mediated by the p38/MSK1 pathway in response to UVB irradiation. J. Biol. Chem..

[5] Batool A., Aashaq S., Andrabi K.I. (2017). Reappraisal to the study of 4E-BP1 as an mTOR substrate—A normative critique. Eur. J. Cell Biol..

[6] Gingras A.C., Gygi S.P., Raught B., Polakiewicz R.D., Abraham R.T., Hoekstra M.F., Aebersold R., Sonenberg N. (1999). Regulation of 4E-BP1 phosphorylation: A novel two-step mechanism. Genes Dev..

[7] Herbert T.P., Tee A.R., Proud C.G. (2002). The extracellular signal-regulated kinase pathway regulates the phosphorylation of 4E-BP1 at multiple sites. J. Biol. Chem..

[8] Johnson G.L., Lapadat R. (2002). Mitogen-activated protein kinase pathways mediated by ERK, JNK, and p38 protein kinases. Science.

[9] Bardwell L. (2006). Mechanisms of MAPK signalling specificity. Biochem. Soc. Trans..

[10] Darling N.J., Balmanno K., Cook S.J. (2017). ERK1/2 signalling protects against apoptosis following endoplasmic reticulum stress but cannot provide long-term protection against BAX/BAK-independent cell death. PLoS ONE.

[11] Roberts P.J., Der C.J. (2007). Targeting the Raf-MEK-ERK mitogen-activated protein kinase cascade for the treatment of cancer. Oncogene.

[12] Cai B., Chang S.H., Becker E.B., Bonni A., Xia Z. (2006). p38 MAP kinase mediates apoptosis through phosphorylation of BimEL at Ser-65. J. Biol. Chem..

[13] Coulthard L.R., White D.E., Jones D.L., McDermott M.F., Burchill S.A. (2009). p38(MAPK): Stress responses from molecular mechanisms to therapeutics. Trends Mol. Med..

[14] Kato H., Nakajima S., Saito Y., Takahashi S., Katoh R., Kitamura M. (2012). mTORC1 serves ER stress-triggered apoptosis via selective activation of the IRE1-JNK pathway. Cell Death Differ..

[15] Verheij M., Ruiter G.A., Zerp S.F., van Blitterswijk W.J., Fuks Z., Haimovitz-Friedman A., Bartelink H. (1998). The role of the stress-activated protein kinase (SAPK/JNK) signaling pathway in radiation-induced apoptosis. Radiother. Oncol. J. Eur. Soc. Ther. Radiol. Oncol..

[16] Estrada Y., Dong J., Ossowski L. (2009). Positive crosstalk between ERK and p38 in melanoma stimulates migration and in vivo proliferation. Pigment. Cell Melanoma Res..

[17] Saxton R.A., Sabatini D.M. (2017). mTOR Signaling in Growth, Metabolism, and Disease. Cell.

[18] Populo H., Lopes J.M., Soares P. (2012). The mTOR signalling pathway in human cancer. Int. J. Mol. Sci..

[19] Albert S., Serova M., Dreyer C., Sablin M.P., Faivre S., Raymond E. (2010). New inhibitors of the mammalian target of rapamycin signaling pathway for cancer. Expert Opin. Investig. Drugs.

[20] Manning B.D., Toker A. (2017). AKT/PKB Signaling: Navigating the Network. Cell.

[21] O’Reilly K.E., Rojo F., She Q.B., Solit D., Mills G.B., Smith D., Lane H., Hofmann F., Hicklin D.J., Ludwig D.L. (2006). mTOR inhibition induces upstream receptor tyrosine kinase signaling and activates Akt. Cancer Res..

[22] Soares H.P., Ni Y., Kisfalvi K., Sinnett-Smith J., Rozengurt E. (2013). Different patterns of Akt and ERK feedback activation in response to rapamycin, active-site mTOR inhibitors and metformin in pancreatic cancer cells. PLoS ONE.

[23] Chresta C.M., Davies B.R., Hickson I., Harding T., Cosulich S., Critchlow S.E., Vincent J.P., Ellston R., Jones D., Sini P. (2010). AZD8055 is a potent, selective, and orally bioavailable ATP-competitive mammalian target of rapamycin kinase inhibitor with in vitro and in vivo antitumor activity. Cancer Res..

[24] Garcia-Echeverria C. (2010). Allosteric and ATP-competitive kinase inhibitors of mTOR for cancer treatment. Bioorganic Med. Chem. Lett..

[25] Chapuis N., Tamburini J., Green A.S., Vignon C., Bardet V., Neyret A., Pannetier M., Willems L., Park S., Macone A. (2010). Dual inhibition of PI3K and mTORC1/2 signaling by NVP-BEZ235 as a new therapeutic strategy for acute myeloid leukemia. Clin. Cancer Res. Off. J. Am. Assoc. Cancer Res..

[26] Feldman M.E., Apsel B., Uotila A., Loewith R., Knight Z.A., Ruggero D., Shokat K.M. (2009). Active-site inhibitors of mTOR target rapamycin-resistant outputs of mTORC1 and mTORC2. PLoS Biol..

[27] Thoreen C.C., Kang S.A., Chang J.W., Liu Q., Zhang J., Gao Y., Reichling L.J., Sim T., Sabatini D.M., Gray N.S. (2009). An ATP-competitive mammalian target of rapamycin inhibitor reveals rapamycin-resistant functions of mTORC1. J. Biol. Chem..

[28] Blaser B., Waselle L., Dormond-Meuwly A., Dufour M., Roulin D., Demartines N., Dormond O. (2012). Antitumor activities of ATP-competitive inhibitors of mTOR in colon cancer cells. BMC Cancer.

[29] Chen Y., Lee C.H., Tseng B.Y., Tsai Y.H., Tsai H.W., Yao C.L., Tseng S.H. (2018). AZD8055 Exerts Antitumor Effects on Colon Cancer Cells by Inhibiting mTOR and Cell-cycle Progression. Anticancer Res..

[30] Willems L., Chapuis N., Puissant A., Maciel T.T., Green A.S., Jacque N., Vignon C., Park S., Guichard S., Herault O. (2012). The dual mTORC1 and mTORC2 inhibitor AZD8055 has anti-tumor activity in acute myeloid leukemia. Leukemia.

[31] Ewald F., Norz D., Grottke A., Bach J., Herzberger C., Hofmann B.T., Nashan B., Jucker M. (2015). Vertical Targeting of AKT and mTOR as Well as Dual Targeting of AKT and MEK Signaling Is Synergistic in Hepatocellular Carcinoma. J. Cancer.

[32] Chen S.M., Guo C.L., Shi J.J., Xu Y.C., Chen Y., Shen Y.Y., Su Y., Ding J., Meng L.H. (2014). HSP90 inhibitor AUY922 abrogates up-regulation of RTKs by mTOR inhibitor AZD8055 and potentiates its antiproliferative activity in human breast cancer. Int. J. Cancer.

[33] Wei F., Zhang Y.D., Geng L., Zhang P., Wang G.Y., Liu Y. (2015). mTOR Inhibition Induces EGFR Feedback Activation in Association with Its Resistance to Human Pancreatic Cancer. Int. J. Mol. Sci..

[34] Bailey S.T., Zhou B., Damrauer J.S., Krishnan B., Wilson H.L., Smith A.M., Li M., Yeh J.J., Kim W.Y. (2014). mTOR inhibition induces compensatory, therapeutically targetable MEK activation in renal cell carcinoma. PLoS ONE.

[35] Hoang B., Benavides A., Shi Y., Yang Y., Frost P., Gera J., Lichtenstein A. (2012). The PP242 mammalian target of rapamycin (mTOR) inhibitor activates extracellular signal-regulated kinase (ERK) in multiple myeloma cells via a target of rapamycin complex 1 (TORC1)/eukaryotic translation initiation factor 4E (eIF-4E)/RAF pathway and activation is a mechanism of resistance. J. Biol. Chem..

[36] Pakos-Zebrucka K., Koryga I., Mnich K., Ljujic M., Samali A., Gorman A.M. (2016). The integrated stress response. EMBO Rep..

[37] Elvira R., Cha S.J., Noh G.M., Kim K., Han J. (2020). PERK-Mediated eIF2alpha Phosphorylation Contributes to The Protection of Dopaminergic Neurons from Chronic Heat Stress in Drosophila. Int. J. Mol. Sci..

[38] Cano-Gonzalez A., Mauro-Lizcano M., Iglesias-Serret D., Gil J., Lopez-Rivas A. (2018). Involvement of both caspase-8 and Noxa-activated pathways in endoplasmic reticulum stress-induced apoptosis in triple-negative breast tumor cells. Cell Death Dis..

[39] Petigny-Lechartier C., Duboc C., Jebahi A., Louis M.H., Abeilard E., Denoyelle C., Gauduchon P., Poulain L., Villedieu M. (2017). The mTORC1/2 Inhibitor AZD8055 Strengthens the Efficiency of the MEK Inhibitor Trametinib to Reduce the Mcl-1/[Bim and Puma] ratio and to Sensitize Ovarian Carcinoma Cells to ABT-737. Mol. Cancer Ther..

[40] Xu D.Q., Toyoda H., Qi L., Morimoto M., Hanaki R., Iwamoto S., Komada Y., Hirayama M. (2018). Induction of MEK/ERK activity by AZD8055 confers acquired resistance in neuroblastoma. Biochem. Biophys. Res. Commun..

[41] Wu P.K., Park J.I. (2015). MEK1/2 Inhibitors: Molecular Activity and Resistance Mechanisms. Semin. Oncol..

[42] Monick M.M., Powers L.S., Gross T.J., Flaherty D.M., Barrett C.W., Hunninghake G.W. (2006). Active ERK contributes to protein translation by preventing JNK-dependent inhibition of protein phosphatase 1. J. Immunol..

[43] Rolli-Derkinderen M., Machavoine F., Baraban J.M., Grolleau A., Beretta L., Dy M. (2003). ERK and p38 inhibit the expression of 4E-BP1 repressor of translation through induction of Egr-1. J. Biol. Chem..

[44] Yanagiya A., Suyama E., Adachi H., Svitkin Y.V., Aza-Blanc P., Imataka H., Mikami S., Martineau Y., Ronai Z.A., Sonenberg N. (2012). Translational homeostasis via the mRNA cap-binding protein, eIF4E. Mol. Cell.

[45] Cope C.L., Gilley R., Balmanno K., Sale M.J., Howarth K.D., Hampson M., Smith P.D., Guichard S.M., Cook S.J. (2014). Adaptation to mTOR kinase inhibitors by amplification of eIF4E to maintain cap-dependent translation. J. Cell Sci..

[46] Banko J.L., Poulin F., Hou L., DeMaria C.T., Sonenberg N., Klann E. (2005). The translation repressor 4E-BP2 is critical for eIF4F complex formation, synaptic plasticity, and memory in the hippocampus. J. Neurosci. Off. J. Soc. Neurosci..

[47] Tsukiyama-Kohara K., Vidal S.M., Gingras A.C., Glover T.W., Hanash S.M., Heng H., Sonenberg N. (1996). Tissue distribution, genomic structure, and chromosome mapping of mouse and human eukaryotic initiation factor 4E-binding proteins 1 and 2. Genomics.

[48] Tamburini J., Green A.S., Bardet V., Chapuis N., Park S., Willems L., Uzunov M., Ifrah N., Dreyfus F., Lacombe C. (2009). Protein synthesis is resistant to rapamycin and constitutes a promising therapeutic target in acute myeloid leukemia. Blood.

[49] Graff J.R., Konicek B.W., Vincent T.M., Lynch R.L., Monteith D., Weir S.N., Schwier P., Capen A., Goode R.L., Dowless M.S. (2007). Therapeutic suppression of translation initiation factor eIF4E expression reduces tumor growth without toxicity. J. Clin. Investig..

[50] Schmieder R., Puehler F., Neuhaus R., Kissel M., Adjei A.A., Miner J.N., Mumberg D., Ziegelbauer K., Scholz A. (2013). Allosteric MEK1/2 inhibitor refametinib (BAY 86-9766) in combination with sorafenib exhibits antitumor activity in preclinical murine and rat models of hepatocellular carcinoma. Neoplasia.

[51] Lim H.Y., Heo J., Choi H.J., Lin C.Y., Yoon J.H., Hsu C., Rau K.M., Poon R.T., Yeo W., Park J.W. (2014). A phase II study of the efficacy and safety of the combination therapy of the MEK inhibitor refametinib (BAY 86-9766) plus sorafenib for Asian patients with unresectable hepatocellular carcinoma. Clin. Cancer Res. Off. J. Am. Assoc. Cancer Res..

[52] Huynh H., Ong R., Goh K.Y., Lee L.Y., Puehler F., Scholz A., Politz O., Mumberg D., Ziegelbauer K. (2019). Sorafenib/MEK inhibitor combination inhibits tumor growth and the Wnt/betacatenin pathway in xenograft models of hepatocellular carcinoma. Int. J. Oncol..

[53] Holt S.V., Logie A., Davies B.R., Alferez D., Runswick S., Fenton S., Chresta C.M., Gu Y., Zhang J., Wu Y.L. (2012). Enhanced apoptosis and tumor growth suppression elicited by combination of MEK (selumetinib) and mTOR kinase inhibitors (AZD8055). Cancer Res..

[54] Fey D., Croucher D.R., Kolch W., Kholodenko B.N. (2012). Crosstalk and signaling switches in mitogen-activated protein kinase cascades. Front. Physiol..

[55] Muranen T., Selfors L.M., Hwang J., Gallegos L.L., Coloff J.L., Thoreen C.C., Kang S.A., Sabatini D.M., Mills G.B., Brugge J.S. (2016). ERK and p38 MAPK Activities Determine Sensitivity to PI3K/mTOR Inhibition via Regulation of MYC and YAP. Cancer Res..

[56] Aguirre-Ghiso J.A., Estrada Y., Liu D., Ossowski L. (2003). ERKMAPK activity as a determinant of tumor growth and dormancy; Regulation by p38(SAPK). Cancer Res..

[57] Gingras A.C., Kennedy S.G., O’Leary M.A., Sonenberg N., Hay N. (1998). 4E-BP1, a repressor of mRNA translation, is phosphorylated and inactivated by the Akt(PKB) signaling pathway. Genes Dev..

[58] Graff J.R., Konicek B.W., Carter J.H., Marcusson E.G. (2008). Targeting the eukaryotic translation initiation factor 4E for cancer therapy. Cancer Res..

[59] She Q.B., Halilovic E., Ye Q., Zhen W., Shirasawa S., Sasazuki T., Solit D.B., Rosen N. (2010). 4E-BP1 is a key effector of the oncogenic activation of the AKT and ERK signaling pathways that integrates their function in tumors. Cancer Cell.

[60] Ducker G.S., Atreya C.E., Simko J.P., Hom Y.K., Matli M.R., Benes C.H., Hann B., Nakakura E.K., Bergsland E.K., Donner D.B. (2014). Incomplete inhibition of phosphorylation of 4E-BP1 as a mechanism of primary resistance to ATP-competitive mTOR inhibitors. Oncogene.

[61] Alain T., Morita M., Fonseca B.D., Yanagiya A., Siddiqui N., Bhat M., Zammit D., Marcus V., Metrakos P., Voyer L.A. (2012). eIF4E/4E-BP Ratio Predicts the Efficacy of mTOR Targeted Therapies. Cancer Res..

[62] Fadden P., Haystead T.A., Lawrence J.C. (1997). Identification of phosphorylation sites in the translational regulator, PHAS-I, that are controlled by insulin and rapamycin in rat adipocytes. J. Biol. Chem..

[63] Lin T.A., Kong X., Saltiel A.R., Blackshear P.J., Lawrence J.C. (1995). Control of PHAS-I by insulin in 3T3-L1 adipocytes. Synthesis, degradation, and phosphorylation by a rapamycin-sensitive and mitogen-activated protein kinase-independent pathway. J. Biol. Chem..

[64] Elia A., Constantinou C., Clemens M.J. (2008). Effects of protein phosphorylation on ubiquitination and stability of the translational inhibitor protein 4E-BP1. Oncogene.

[65] De Benedetti A., Graff J.R. (2004). eIF-4E expression and its role in malignancies and metastases. Oncogene.

[66] Jeong M., Cho J., Cho W.S., Shin G.C., Lee K. (2009). The Glucosamine-Mediated Induction of CHOP Reduces the Expression of Inflammatory Cytokines by Modulating JNK and NF-kappa B in LPS-Stimulated RAW264.7 Cells. Genes Genom..

[67] Han J., Back S.H., Hur J., Lin Y.H., Gildersleeve R., Shan J., Yuan C.L., Krokowski D., Wang S., Hatzoglou M. (2013). ER-stress-induced transcriptional regulation increases protein synthesis leading to cell death. Nat. Cell Biol..

[68] Shin J.I., Jeon Y.J., Lee S., Lee Y.G., Kim J.B., Lee K. (2019). G-Protein-Coupled Receptor 120 Mediates DHA-Induced Apoptosis by Regulating IP3R, ROS and, ER Stress Levels in Cisplatin-Resistant Cancer Cells. Mol. Cells.

[69] Shin J.I., Jeon Y.J., Lee S., Lee Y.G., Kim J.B., Kwon H.C., Kim S.H., Kim I., Lee K., Han Y.S. (2018). Apoptotic and Anti-Inflammatory Effects of Eupatorium japonicum Thunb. in Rheumatoid Arthritis Fibroblast-Like Synoviocytes. BioMed Res. Int..

[70] Liang S., Guo R., Zhang Z., Liu D., Xu H., Xu Z., Wang X., Yang L. (2013). Upregulation of the eIF4E signaling pathway contributes to the progression of gastric cancer, and targeting eIF4E by perifosine inhibits cell growth. Oncol. Rep..

